# Effects of 1.8 GHz Radiofrequency Fields on the Emotional Behavior and Spatial Memory of Adolescent Mice

**DOI:** 10.3390/ijerph14111344

**Published:** 2017-11-05

**Authors:** Jun-Ping Zhang, Ke-Ying Zhang, Ling Guo, Qi-Liang Chen, Peng Gao, Tian Wang, Jing Li, Guo-Zhen Guo, Gui-Rong Ding

**Affiliations:** 1 Department of Radiation Biology, Faculty of Preventive Medicine, Fourth Military Medical University, 169# Chang Le West Road, Xi’an 710032, China; xp10260641@163.com (J.-P.Z.); zhangky@fmmu.edu.cn (K.-Y.Z.); guolingclover@163.com (L.G.); chenqiliang23@163.com (Q.-L.C.); scuwangtian1989@sina.cn (T.W.); jingli@fmmu.edu.cn (J.L.); 2 Department of Radiation Medicine, Faculty of Preventive Medicine, Fourth Military Medical University, 169# Chang Le West Road, Xi’an 710032, China; patriciag@126.com (P.G.); guozhen@fmmu.edu.cn (G.-Z.G.)

**Keywords:** RF field, anxiety-like behavior, depression-like behavior, spatial memory, amino acid neurotransmitters

## Abstract

The increasing use of mobile phones by teenagers has raised concern about the cognitive effects of radiofrequency (RF) fields. In this study, we investigated the effects of 4-week exposure to a 1.8 GHz RF field on the emotional behavior and spatial memory of adolescent male mice. Anxiety-like behavior was evaluated by open field test (OFT) and elevated plus maze (EPM) test, while depression-like behavior was evaluated by sucrose preference test (SPT), tail suspension test (TST) and forced swim test (FST). The spatial learning and memory ability were evaluated by Morris water maze (MWM) experiments. The levels of amino acid neurotransmitters were determined by liquid chromatography-mass spectrometry (LC-MS). The histology of the brain was examined by hematoxylin-eosin (HE) staining. It was found that the depression-like behavior, spatial memory ability and histology of the brain did not change obviously after RF exposure. However, the anxiety-like behavior increased in mice, while, the levels of γ-aminobutyric acid (GABA) and aspartic acid (Asp) in cortex and hippocampus significantly decreased after RF exposure. These data suggested that RF exposure under these conditions do not affect the depression-like behavior, spatial memory and brain histology in adolescent male mice, but it may however increase the level of anxiety, and GABA and Asp were probably involved in this effect.

## 1. Introduction

With the widespread use of mobile phones in modern life, adolescents are exposed to radiofrequency (RF) fields from mobile phones and base stations much more frequently than ever. According to one report, 90% of adolescents have a mobile phone and about 40% of them are considered as heavy mobile phone users [1]. Therefore, this has raised public concerns about the potential health hazard of RF fields on adolescents. 

RF field is a kind of electromagnetic radiation with frequencies ranging from 100 kHz to 300 GHz, which is used extensively in wireless communications, such as mobile phones, radios and televisions. The RF field produced by mobile phones and base stations is mainly at the frequencies of 800–2000 MHz [2]. It was reported that the average field density of the mobile phone antenna nearby was about 200 μW/cm^2^–300 μW/cm^2^ [3], exceeding the limit stipulated by the national standard in China (40 μW/cm^2^) [4].

The head receives much more RF energy than any other part of body because the mobile phone is usually held at the ear during communication. Whether RF exposure results in brain dysfunction, has become a major topic of investigation [5]. Based on the evidence from epidemiological and laboratory studies, RF fields in the 30 kHz–300 GHz frequency range were classified as a possible human carcinogen (Group 2B) by the International Agency for Research on Cancer (IARC) in 2011 [6]. In addition, it was reported that exposure to RF fields could cause insomnia, headache, tinnitus, fatigue, cognitive disturbances, dysesthesias (abnormal sensation), dizziness, and depression [7,8,9]. 

In terms of the effects of RF field on brain function, Wang found that although single exposure to 1800 MHz RF field for 30 min with specific absorption rates (SARs) in brain at 2.42, 2.86 and 3.3 W/kg did not affect spontaneous locomotor activity, it increased recognition memory in mice [10]. In addition, Bouji reported that after exposing the heads of the animals to 900 MHz RF field for 1 month (SAR 6 W/kg, 45 min/day), the spatial memory, emotional memory and locomotor activity did not change, but the anxiety-related behavior decreased in male senescent rats [11]. Arendash reported that exposure to 918 MHz RF field (SAR: 0.25 W/kg, 1 h/day for 4–7 months) could protect against and reverse cognitive impairment in Alzheimer’s disease mice [12]. However, some adverse effects were also reported. Aldad found that exposure to 800–1900 MHz-rated cellular telephones resulted in memory impairments in fetal mice [13]. In addition, Daniels reported that although exposure to 840 MHz RF field (power density: 60 μW/m^2^, 3 h/day for 13 days) had no effect on spatial memory and brain morphology, it decreased the locomotor activity in rats [14]. 

Over all, the effects of RF exposure on animal behaviors and memory remain inconclusive. In present study, we explored the effects of 4-week exposure to 1.8 GHz RF field on the emotional behavior and spatial memory in adolescent male mice.

## 2. Materials and Methods

### 2.1. Animals and Groups 

A total of 70 male C57BL/6 mice (4 weeks old), purchased from the Experimental Animal Center of Fourth Military Medical University, were randomly divided into RF exposure group and sham exposure group. The animals were whole body exposed or sham exposed to RF field for 4 weeks, 6 h/day. After exposure, mice in both RF group and sham group were divided into three sub-groups, respectively, according to the detection endpoints. Animals in the first sub-group (n = 10) were used for open field tests (OFT), sucrose preference tests (SPT) and tail suspension tests (TST), while animals in the second sub-group (n = 10) were used for the elevated plus maze (EPM) and forced swim test (FST), and animals in the third sub-group (n = 15) were used for the Morris water maze (MWM) assay. All animals were kept in a controlled environment (25 ± 2 °C, 12-h light and 12-h dark cycle), and they were handled daily and allowed to acclimate to the laboratory for 1 week before the start of the experiments. All experiments reported in this study were conducted according to an experimental protocol approved by the Animal Use and Care Committee for Research and Education of Fourth Military Medical University (20160105, Xi’an, China).

### 2.2. Radiofrequency Field Exposure 

The 1.8 GHz RF exposure system (East China Normal University, Shanghai, China), which has been described elsewhere [10], is comprised of a RF signal source, a movable loading platform and a computer. The mice in the exposure group were placed freely in a plastic box with bedding (35 × 24 × 24 cm with small ventilation holes on the walls). Mice were exposed to 1.8 GHz frequency field for 28 days, 6 h/day (9:00 AM to 3:00 PM). During exposure, the animals had access to food and water. The SAR of whole body and brain were approximately 2.7 W/kg and 2.2 W/kg at a distance of 1 m from the antenna. Since the incidence and polarization of free movement mice kept changing during exposure, the deviation between the average SAR value and the maximum and the minimum of SAR was about 8%. The power density in this study was 530 μW/cm^2^, as measured by an electromagnetic field meter (PMM8053A, PMM Costruzioni Electtroniche Centro Misure Radio Electriche S.r.l., Milan, Italy). A dosimetry assessment similar to that of a previous report [10] was used in this study. Briefly, the average SARs of whole body and brain were calculated using XFDTD7.2.3 (Remcom Inc., State College, PA, USA) and the reference electric constants of the tissues were derived from http://transition.fcc.gov/oet/rfsafety/dielectric.html. During exposure, the mice were awake and quiet and they did not show any abnormal physical responses to the RF signals. Sham group mice underwent exposure in the same way as RF group mice but the control system was turned off.

**Figure 1:**
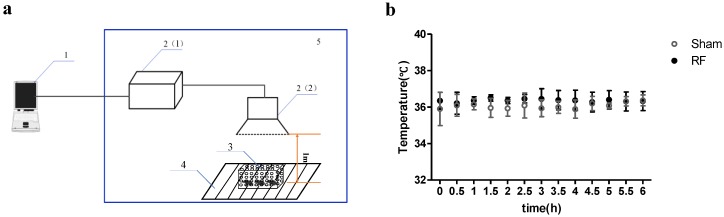
Schematic diagram of experimental set-up of the RF-EMR-exposure system (**a**) 1. Control system: a computer; 2. Microwave signal source. (1). Microwave source; (2). Horn antenna; 3. Experiment animals were placed at a plastic box (35 × 24 × 24 cm) with many vents on their walls; 4. Experiment platform; 5. Shielded room. Time course of changes in mice surface body temperature during 1.8 GHz RF field exposure (**b**). Data were represented as the mean ± SD. N = 6 for each group (within group: df = 12, F = 0.323, *P* = 0.984; between groups: df = 1, F = 2.721, *P* = 0.130); RF: radiofrequency.

During exposure, we simultaneously measured the surface body temperature of mice with an infrared thermal camera (Fluke Ti400 Thermal Imager, Fluke Corporation, Everett, WA, USA). It was found that exposure to 2.70 W/kg RF field did not obviously affect the surface body temperature of mice (Figure 1), which suggested that no gross thermal effects were involved. Additionally, the air temperature of the exposure cage with a temperature hygrometer was measured and the results showed that air temperature difference did not exceed 0.1 °C during 6 h exposure.

### 2.3. Behavior Assessment

24 h after 28-day RF exposure (29th day) OFT, EPM, TST and FST were assessed. SPT was conducted from 25th (exposure day) to 29th day, and MWM was carried out from 24th (exposure day) to 29th day. Mice were habituated to the testing room for more than half an hour before the behavioral test.

#### 2.3.1. Open Field Test (OFT)

OFT is commonly used to assess locomotor activity, exploratory and anxiety-related behavior in rodents. The open field apparatus was consisted of a clear plexiglass box (23 × 23 × 35 cm) with a white floor. Mice were placed in the center of the apparatus to measure the total distance moved for 5 min under a dim light (20 lx) by a computer-controlled tracking system (Mobile Datum Information Technology Co., Ltd., Shanghai, China). The accumulative distance and the time spent in the central area of the apparatus were also measured to evaluate the level of anxiety. After each individual test, apparatus was cleaned with alcohol solution.

#### 2.3.2. Elevated Plus Maze (EPM)

EPM was performed to evaluate the anxiety-like behaviors based on the natural aversion of rodents for open and elevated areas. The instrument was consisted of two closed arms and two open arms, which were made of acryl material and placed at the height of 50 cm above the floor. At the beginning of each test, the mouse was placed in the central area facing open arms and was allowed to explore freely for 5 min. The animals’ movements track was recorded with a digital camera and analyzed by software (Mobile Datum Information Technology Co., Ltd., Shanghai, China). Total entries into the arms, percentage of time spent in open arms, and percentage of entries in open arms were analyzed. The maze was cleaned before each test with alcohol solution.

#### 2.3.3. Sucrose Preference Test (SPT)

SPT was generally acknowledged as one of the most effective ways to detect the depression-like behavior according to the natural sugar water preference of rodents. The test lasted for five days and every mouse was fed in single cage during the test. Each mouse was given two bottles of water in the first day afternoon. In the second day afternoon within the compartment, the water in one of two bottles was changed to 2.5% sugar water and kept there for next two days. In the fourth day afternoon, 2.5% sugar water was replaced by 1% sugar water, and then, the intake of the 1% sugar water and normal water were measured in the fifth day afternoon (for 24 h). In order to avoid the preference of position, two bottles’ positions were exchanged in the intermediate time. The water preference index was calculated according to 100% × (the sugar water intake)/(the normal water intake + the sugar water intake), which was used to evaluate the depression-like behavior of each animal.

#### 2.3.4. Tail Suspension Test (TST)

TST is widely used to measure depression-like behavior in rodents. The apparatus is composed of five coordinate compartments with three-walled rectangular (55 cm height × 15 cm width × 11.5 cm depth). Mice were suspended with the tail within the compartment of small plastic cylinders and the approximate distance between the mouse's nose and the apparatus floor was 20–25 cm. The total duration of immobility was measured in seconds. Notably, small movements confined to the front legs instead of the hind legs were counted as immobility. Additionally, oscillations and pendulum like swings that were due to the momentum gained during the earlier mobility bouts also counted as immobility. Every mouse was tested for 6 min and the duration of immobility was measured [15]. All data were analyzed by two observers who were blind to the group assignment of animals.

#### 2.3.5. Forced Swim Test (FST)

FST was performed with a glass cylinder (20 cm diameters × 30 cm height) filled with warm water (23–25 °C). The water level was 15 cm from the bottom so that the mice were not able to touch the bottom of the tank, either with their feet or tails, during the swimming test. A total of 6 min of testing was carried out and only the last 4 min of the test were analyzed. The mobility time was measured in seconds only when the mice do any movements other than those necessary to balance the body and keep the head above the water. After each test, the water in glass cylinder was changed with the fresh water at the same temperature. Mice were dried by a heat lamp (the temperature of the lamp did not exceed 32 °C) after the test [16]. All data were analyzed by two observers who were blind to the group assignment of animals.

#### 2.3.6. Morris Water Maze (MWM)

MWM was used to measure the ability of spatial learning and memory. The procedure was reported in detail by Voorhees and Williams in 2006 [17]. In brief, the water maze was a circular tank (180 cm in diameter and 60 cm in height) located in a room containing a variety of cues. The pool was filled with opaque water that was made by adding white food additives and the temperature was maintained at 22–25 °C. The tank was divided into four quadrants and an invisible platform was 1 cm below the surface of the water. There were four trials per day for each mouse during the 5 days training. For each trial, the mouse was randomly put into the water in one of the quadrants with its head facing the tank wall. Each mouse was allowed to swim freely until it came across and climbed on the platform and stayed there for 10 s. In case a mouse did not reach the platform after 60 s swimming, it was gently hand-guided to the platform. 24 h after 5th training test, the probe test was performed. Before probe test, the platform was removed, and then the mouse was allowed to swim for 60 s. All trials were processed on line by a video track tracking system (Mobile Datum Information Technology Co., Ltd., Shanghai, China). The escape latency, the time spent in the target quadrant and the times crossing the platform were used to evaluate the mice’s spatial learning and memory ability.

### 2.4. Body and Organ Weight Measurements 

In this study, all animals’ body weight was measured. No animals got sick or died during the experiments. The body weight was measured about every 4 days during the experiment (from 0th to 30th day, 48 h after 28-day RF exposure). At the end of the behavioral experiments (30th day), 50 mice were deeply anaesthetized by sodium pentobarbital (60 mg/kg) and then the brains were isolated and weighed immediately. Subsequently, the cortex and hippocampus were removed separately, washed with cold phosphate buffered saline (PBS, pH 7.4), dried with filter paper, weighted and stored at −80 °C for neurotransmitters and acetylcholinesterase (AchE) detection later. Growth curve, weight gain, the ratio of the hippocampus weight to the brain weight, the ratio of the hippocampus weight to the body weight and the ratio of the brain weight to the body weight were calculated.

### 2.5. Hematoxylin and Eosin (HE) Staining 

On the 30th day, 20 mice were anaesthetized as previously described and fixed via cardiac perfusion with 4% paraformaldehyde after flushing out the red blood cells with PBS. The whole brains were isolated and fixed in 4% paraformaldehyde for 1 day. The brains were then dehydrated in ethanol, defatted in xylene, embedded in paraffin, and serially sectioned at 5 μm thicknesses by using a rotary microtome (Leica RM2135, Leica Biosystems, Heidelberg, Germany). Subsequently, the sections were stained with HE according to procedure which has been reported [18]. The digital photograph of hippocampal CA1, CA3 and amygdala were observed by Leica DMI4000B (Leica Biosystems, Heidelberg, Germany) and a Hamamatsu Nano Zoomer scan SQ1.0 (Hamamatsu Photonics, Shizuoka, Japan) with NDP, respectively. 

### 2.6. Amino Acid Neurotransmitters Detected by LC-MS

The samples (10 hippocampi and 10 cortices from each group) were homogenized with 0.5 mL of 1% formic acid solution prepared in cold acetonitrile (−20 °C) using a homogenizer device (Leica Biosystems, Heidelberg, Germany), then the supernatant were extracted after centrifugation at 12,000 rpm for 20 min at 4 °C. After that, the level of amino acid neurotransmitters such as Asp, GABA, glutamic acid (Glu) and glycine (GLY) were detected by liquid chromatography-mass spectrometry (LC-MS) (AB SCIEX QTRAP® 4500, SCIEX, Framingham, MA, USA) as reported [19]. This equipment was provided by the Centers for Disease Control and Prevention (CDC) of Xi’an.

### 2.7. Acetylcholinesterase (AchE) Activity Detection 

The tissue (seven hippocampi and seven cortices from each group) were used to detect the activity of AchE by a commercial kit (Acetylcholinesterase Assay Kit, DACE-100, BioAssay Systems, Hayward, CA, USA). The samples were homogenized with 1.5 mL cold PBS (pH 7.4) by a homogenate device and the supernatant were collected after centrifugation at 3000 rpm for 20 min at 4 °C. After that, the supernatant were used to detect the activity of AchE according to the manufacturer’s protocol.

### 2.8. Statistical Analysis

All results were presented as mean ± standard deviation (SD). Data analysis was performed using SPSS 17.0 software (SPSS Inc., Chicago, IL, USA) by the individual blinded to group of exposure. Repeated measures analysis of variance (ANOVA) was conducted on data which was recorded in blocks across time, and student *t*-test was used on the other data. It was considered statistically significant only when *P* value less than 0.05. 

## 3. Results

### 3.1. Effects of RF Exposure on Anxiety-Like Behaviors 

**Figure 2:**
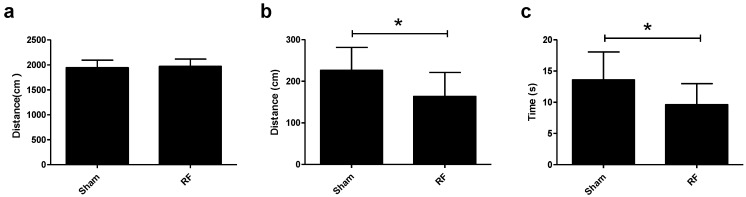
Effects of RF field exposure on anxiety-like behaviors. (**a**) Total distance traveled in OFT (t = 0.385, df = 18, *P* = 0.705), (**b**) accumulative distance in the center area in OFT (t = 2.5, df = 18, *P* = 0.022), (**c**) time spent in the central area in OFT (t = 2.548, df = 18, *P* = 0.020). All data are presented as mean ± SD, * *P* < 0.05, compared with the sham group.

The anxiety-like behaviors of mice were evaluated by OFT and EPM after RF exposure. OFT results showed that there were no significant differences in accumulative total distance traveled between the sham group and RF group (Figure 2a). However, the accumulative distance in the center area and the time spent in the central area decreased significantly in the RF group, compared with the sham group (Figure 2b,c), which indicated that 4-week RF exposure could increase the animals’ anxiety-like behavior.

**Figure 3:**
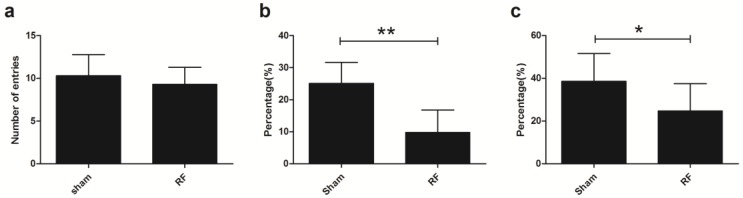
Effects of RF field exposure on anxiety-like behaviors. (**a**) Number of entries into the arms (open arms and closed arms) in EPM (t = 0.988, df = 18, *P* = 0.336), (**b**) percentage of the total time spent in the open arms in EPM (t = 5.030, df = 18, *P* < 0.0001), (**c**) percentage of the entries into the open arms in EPM (t = 2.411, df = 18, *P* = 0.027). All data are presented as mean ± SD, * *P* < 0.05, ** *P* < 0.01, compared with the sham group.

In addition, EPM results showed that there were no significant differences in the number of total entries into the arms between the sham group and RF group, which indicated that the locomotor activity in the mice did not change after RF exposure (Figure 3a). However, the percentage of the total time spent in the open arms and the percentage of the entries into the open arms decreased significantly in the RF group, compared with the sham group (Figure 3b,c). These results were consistent with that of OFT.

### 3.2. Effects of RF Exposure on Depression-Like Behaviors 

**Figure 4:**
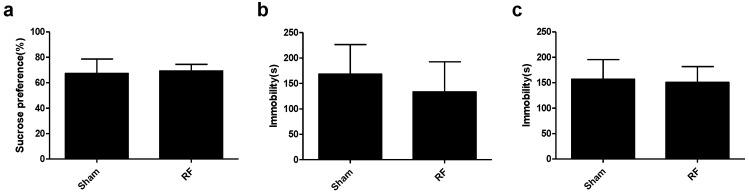
Effects of RF field exposure on depression-like behaviors. (**a**) the index of the SPT (t = 0.501, df = 18, *P* = 0.622), (**b**) the immobility time of the TST (t = 1.438, df = 18, *P* = 0.168), (**c**) the immobility time of the FST (t = 0.417, df = 18, *P* = 0.682). All data are presented as mean ± SD. No significant difference was observed between the sham and RF groups (*P* > 0.05).

In this study, SPT, TST and FST were performed to evaluate the depression-like behaviors after exposing the mice to RF field for 4 weeks. SPT results showed that there were no significant differences in the sucrose preference indices between the sham group and RF group (Figure 4a). Similarly, TST and FST results showed that there were no significant differences in the immobility time between the sham group and the RF group (Figure 4b,c). These results indicated that 4-week RF exposure could not increase depression-like behaviors in mice.

### 3.3. Effects of RF Exposure on the Spatial Learning and Memory

**Figure 5:**
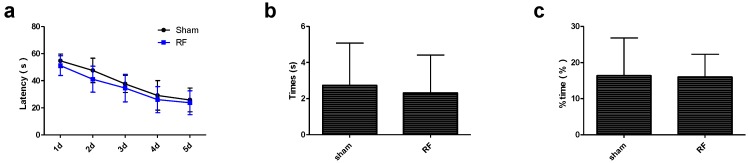
Effects of RF field exposure on the spatial learning and memory. (**a**) the escape latency in training phase (within group: df = 4, F = 68.17, *P* < 0.001; between groups: df = 1, F = 3.185, *P* = 0.085), (**b**) the number of times crossing the platform area in probe phase (t = 0.290, df = 18, *P* = 0.775), (**c**) the time spent in the target quadrant in probe phase (t = 0.127, df = 18, *P* = 0.900). All data are presented as mean ± SD. No significant difference was observed between the sham and RF groups (*P* > 0.05).

It was found that the escape latency in both the sham and RF groups decreased gradually as the training time was extended. However, there were no significant differences in the escape latency during the training phase between the sham group and RF group (Figure 5a). Similarly, there were no significant differences in the number of times crossing the platform and the time spent in the target quadrant between the sham group and RF group during the probe phase (Figure 5b,c). These data indicated that RF exposure had no effects on the spatial learning and memory ability in mice.

### 3.4. Effect of RF Exposure on Body and Organ Weight

**Figure 6:**
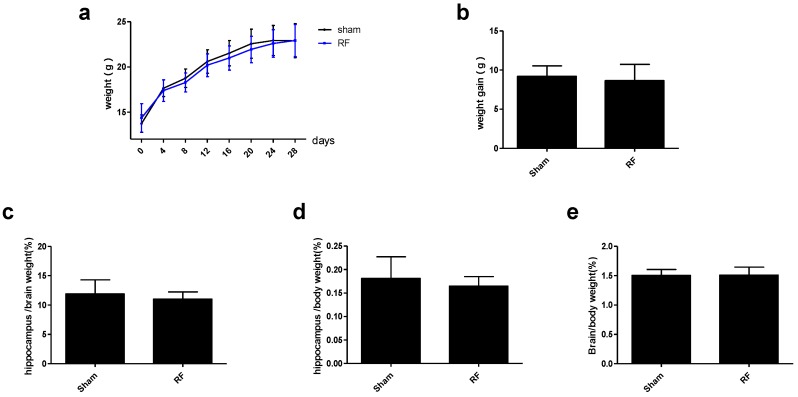
Effects of RF exposure on body and organ weight. (**a**) growth curve (within group: df = 7, F = 657.019, *P* < 0.001; between groups: df = 1, F = 0.251, *P* = 0.619), (**b**) weight gain (t = 1.3, df = 68, *P* = 0.198), (**c**) the ratio of the hippocampus weight to the brain weight (t = 1.615, df = 48, *P* = 0.113), (**d**) the ratio of the hippocampus weight to the body weight (t = 1.751, df = 48, *P* = 0.086), (**e**) the ratio of the brain weight to the body weight (t = 0.145, df = 48, *P* = 0.886). The data are presented as mean ± SD. No significant difference was observed between the sham and RF groups (*P* > 0.05).

As shown in Figure 6a, the body weight in both the sham and RF group increased as time was extended. However, there were no significant differences between the sham group and RF group. Furthermore, the weight gain (weight on 30th day minus weight on 0th exposure day (Figure 6b) showed that there was no difference in weight between the sham group and RF group. Similarly, the ratio of the hippocampus weight to the brain weight (Figure 6c), the ratio of the hippocampus weight to the body weight (Figure 6d), the ratio of the brain weight to the body weight (Figure 6e) did not change obviously in the RF group, compared with sham group. These data indicated that RF exposure had no effect on body and organ (brain, hippocampus) weight.

### 3.5. Effect of RF Exposure on the Histology of the Brain

**Figure 7:**
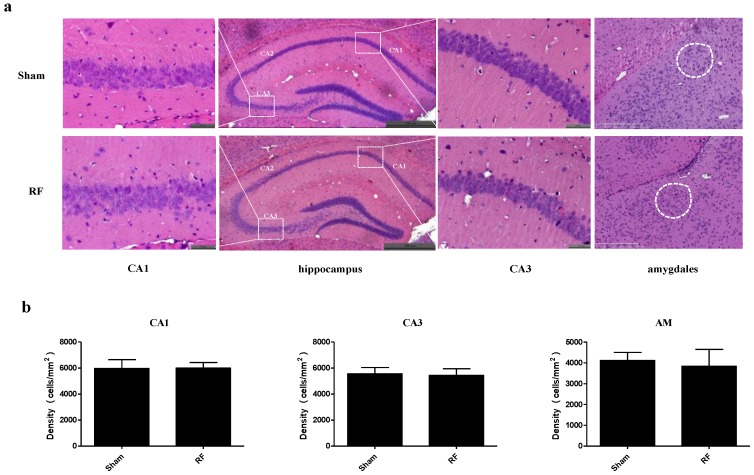
Effects of RF field exposure on the histology of brain. (**a**) The histology of hippocampus (CA1, CA3) and amygdala (AM) examined by HE staining. (**b**) The morphometry analysis of CA1, CA3 and AM (CA1: t = 0.156, df = 18, *P* = 0.878; CA3: t = 0.545, df = 18, *P* = 0.593; AM: t = 1.000, df = 18, *P* = 0.333). Enlargements (CA1: scale bar, 50 μm; hippocampus: scale bar, 500 μm; CA3: scale bar, 50 μm; amygdala: scale bar, 250 μm;) No significant difference was observed between the sham and RF groups (*P* > 0.05).

HE staining results showed no obvious histological and morphometric differences of CA1 and CA3 and amygdala (AM) between the RF group and the sham group (Figure 7).

### 3.6. Effects of RF Exposure on the Levels of Neurotransmitters in Hippocampus and Cortex

**Figure 8:**
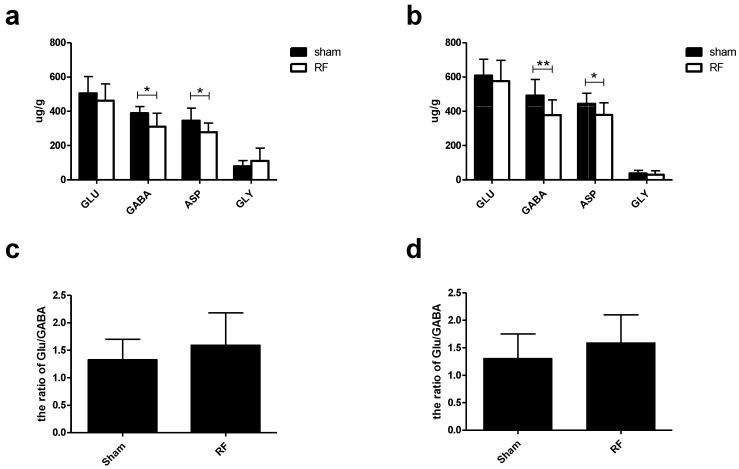
Effects of RF exposure on the levels of four amino acid neurotransmitters in hippocampus and cortex. (**a**) hippocampus (Glu: t = 0.915, df = 16, *P* = 0.374; GABA: t = 2.673, df = 16, *P* = 0.017; ASP: t = 2.266, df = 16, *P* = 0.038; Gly: t = 1.132, df = 16, *P* = 0.275), (**b**) cortex (Glu: t = 0.659, df = 18, *P* = 0.518; GABA: t = 2.939, df = 18, *P* = 0.009; ASP: t = 2.173, df = 18, *P* = 0.043; Gly: t = 0.889, df = 17, *P* = 0.386), (**c**) the ratio of Glu/GABA in hippocampus (t = 1.146, df = 16, *P* = 0.269), (d) the ratio of Glu/GABA in cortex (t = 1.342, df = 18, *P* = 0.196). The data are presented as mean ± SD, * *P* < 0.05, ** *P* < 0.01, compared with the sham group.

LC-MS results showed no obvious differences in the levels of Glu and Gly in hippocampus and cortex between the sham and RF groups. However, the levels of GABA and Asp in hippocampus and cortex were significantly decreased in the RF group, compared with the sham group (Figure 8a,b). In addition, the ratio of Glu/GABA in hippocampus and cortex did not change after RF exposure (Figure 8c,d). 

### 3.7. Effects of RF Exposure on the Activity of AchE in Hippocampus and Cortex

**Figure 9:**
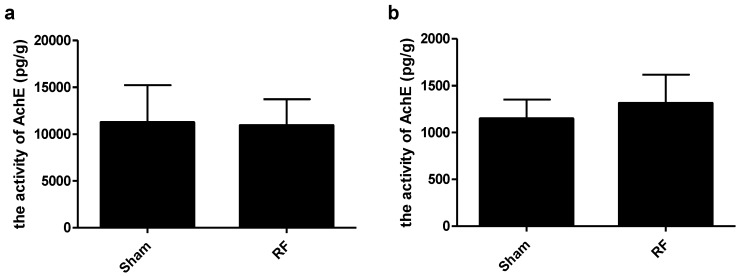
Effects of RF exposure on the activity of AchE in hippocampus and cortex. (**a**) hippocampus (t = 0.172, df = 12, *P* = 0.867), (**b**) cortex (t = 1.178, df = 12, *P* = 0.262). No significant difference was observed between the sham and RF groups (*P* > 0.05).

Acetycholine (Ach) plays an important role in learning and memory modulation. As a necessary hydrolase for Ach, AchE in hippocampus and cortex was measured after RF exposure. It was found that the activity of AchE in hippocampus and cortex did not change after RF exposure, compared with the sham group (Figure 9).

## 4. Discussion

In this study, a sequence of tests was performed to evaluate the effects of RF exposure on the emotional behavior and spatial memory in adolescent mice. Firstly, we evaluated the depression-like behavior by SPT, TST and FST in mice after RF exposure. It was found that the depression-like behavior in mice did not increase after RF exposure. Kumar et al. similarly reported that exposure to chronic modulated microwaves (2.45 GHz, power density: 0.029 mW/cm^2^, SAR: 0.019 W/Kg with sinusoidal modulation of 400 Hz) for 2 h each day for 1 month had no effect on depression-like behavior in mice. However, they also reported that exposure to non-modulated microwave (2.45 GHz, power density: 0.033 mW/cm^2^, SAR: 0.023 W/Kg) for the same period caused depression-like behavior in adult male Swiss mice [20]. Kumar’s findings suggested that non-modulated microwave might have much stronger effects on emotional behavior than modulated microwaves. Nevertheless, in this study, a non-modulated RF field was also used, and no effect on depression-like behavior was found. In addition, it was reported that exposure to 9.417 GHz RF field (power intensity: 200 V/m, SAR: 2.0 W/kg) for 12 h per day during pregnancy days 3.5–18 in mice decreased depression related behavior [21]. The contradictory results could be due to the strain, the age and the gender of animals, as well as the parameters of the electromagnetic fields used in the study. 

Regarding the effects of RF exposure on anxiety-like behavior, the results remain controversial. Bouji reported that exposure to RF field (900 MHz, SAR 6 W/kg, 45 min/day for 1 month) decreased anxiety-related behaviors in both young and aged rats [11]. Kumlin found that exposure to RF field (900 MHz, SAR 9W/kg) for 2 h/day, 5 days per week (exposure from the age of 21 days to the age of 8 weeks) did not increase the level of anxiety in rats [22]. Similarly, Júnior’s result showed that continuous exposure to a 1.8 GHz Global System for Mobile (GSM) cell phone (25-s phone calls in every 2 min, average electric field intensity when the phone was connected, 2.0 V/m, disconnected, 0.1 V/m) for 3 days did not change anxiety-like behavior in rats [23]. In addition, Barthelemy found that exposure to 900 MHz RF field (15 min at 0, 1.5, or 6 W/kg, or for 45 min at 6 W/kg) had no effect on anxiety-like behavior in rats [24]. However, Saikhedkar found that after exposing rats to 900 MHz RF field (0.9 W/kg) for 15 days (4 h/day), the level of anxiety increased [25], which was consistent with our results. The reason of the controversial results may due to the different experimental conditions, such as exposure devices, exposure parameters (power density, duration, exposure time), animals and so on. 

The water maze is one of the most frequently used methods to evaluate the ability of spatial learning and memory, which is first reported in 1984 by Morris [26]. In this study, the MWM results showed that exposure to 1.8 GHz RF field did not change the spatial learning and memory in mice. Similar results were reported by other research groups. For example, Sienkiewicz reported that exposure to 900 MHz RF field for 45 min each day for 10 days (SAR: 0.05 W/kg) had no effect on spatial learning and memory abilities in mice [27]. In addition, Klose found that exposure to 900 MHz RF field (SAR: 0.7, 2.5 and 10 W/kg) for 2 h /day, 5 days/week had no effect on spatial learning and memory abilities in juvenile, adult and presenile rats [28]. However, there are some positive results reported considering the cerebral effects of RF field. Wang found that single and high-power RF field exposure (1.8 GHz, 3.3 W/kg) for 30 min increased the recognition memory ability in mice [10]. Jeong reported that chronic exposure to RF-EMF (1950 MHz, SAR 5 W/kg, 2 h/day, 5 days/week) for 8 months reduced accumulation of Aβ and impairments of non-spatial and spatial memory in an Alzheimer’s disease mouse model [29]. Similarly, Arendash found that exposure to long-term, high-frequency RF field (918 MHz, 0.25 W/kg, 1 h/day for 4–7 months) protected against and reversed cognitive impairment in Alzheimer’s disease mouse model [12]. 

Till now, most of the procedures used in bioelectromagnetic research fields were not pharmacologically validated. With commonly accepted pharmacologically validated procedures, it is essential to (pharmacologically) validate all procedures before the exposure and make the experimental results better replicable by others.

The behavior of animals is regulated by the central nervous system. The hippocampus and amygdala are supposed to be associated with anxiety-like behavior and depression-like behavior [30,31], and the hippocampus is supposed to play an important role in learning and memory [32]. Our result showed that the histology of hippocampus and amygdala did not change in RF group, compared with sham group, which was consistent with the results of SPT, TST, FST and MWM. The result indicated that RF field had no effect on depression-like behavior and spatial learning and memory ability. 

It was reported that levels of amino acid neurotransmitters such as Glu (an excitatory neurotransmitter) and GABA (an inhibitory neurotransmitter) are related to behavior [33,34,35], especially the emotional behavior [36]. In addition, the ratio of Glu to GABA [37] and the activity of AchE [38,39] were reported to play an important role in learning and memory ability. To confirm the behavior results and to explore the possible mechanisms, the levels of neurotransmitters and the activity of AchE were examined in mice brain. Our results showed that the value of Glu/GABA and the activity of AchE in hippocampus and cortex did not change in RF group, compared with sham group, which was consistent with the results of MWM. It was reported that GABA in mice brain, plays a key role in the modulation of anxiety response [40,41,42,43], and in this study, we found that the level of GABA in mice brain decreased significantly after RF exposure. Considering the OFT and EPM results, we speculated that the GABA probably was involved in RF induce anxiety in mice. Additionally, it was found that the level of Asp in mice brain significantly changed after RF exposure. Regarding the relationship between the behavior and Asp remains unclear.

In the present study, brain SAR 2.2 W/kg was selected based on the 2.0 W/kg limit by the International Commission on Nonionizing Radiation Protection (ICNIRP) and Institute of Electrical and Electronics Engineers (IEEE) [44,45]. It was found that after 6 h RF field exposure, the temperature of mice surface body did not change obviously compared with sham group, which suggested that no gross thermal effects were involved in RF-induced anxiety behavior in mice. 

## 5. Conclusions

4-week exposure to 1.8 GHz RF field had no significant effect on depression-like behavior, spatial learning and memory ability or the histology of brain in adolescent male mice. However, it may increase the level of anxiety, and amino acid neurotransmitters such as GABA might be involved.

## References

[1] Petitdant N., Lecomte A., Robidel F., Gamez C., Blazy K., Villégier A. (2016). Cerebral radiofrequency exposures during adolescence: Impact on astrocytes and brain functions in healthy and pathologic rat models. Bioelectromagnetics.

[2] Chen G., Xu Z., Jiang H. (2005). In vitro study on the relationship between radiofrequency electromagnetic field of mobile phone and health. Chin. J. Prev. Med..

[3] Qu B., Wang Y., Xing B., Guo H., Liu J., Zhang Y., Sun G. (2008). Effects of electromagnetic radiation on neurobehavioral and cognitive ability of mobile phones. Chin. J. Radiol. Health.

[4] Ministry of Environmental Protection of the People’s Republic of China, General Administration of Quality Supervision, Inspection and Quarantine of the People’s Republic of China (2014). Controlling limits for electromagnetic environment. GB8702-2014.

[5] Scientific Committee on Emerging Newly Identified Health Risks (2015). Opinion on potential health effects of exposure to electromagnetic fields. Bioelectromagnetics.

[6] International Agency for Research on Cancer (2013). Non-Ionizing Radiation, Part 2: Radiofrequency Electromagnetic Fields. IARC Monographs on the Evaluation of Carcinogenic Risks to Humans.

[7] Calvente I., Pérez-Lobato R., Núñez M., Ramos R., Guxens M., Villalba J., Olea N., Fernández M.F. (2016). Does exposure to environmental radiofrequency electromagnetic fields cause cognitive and behavioral effects in 10-year-old boys. Bioelectromagnetics.

[8] Lamech F. (2014). Self-reporting of symptom development from exposure to radiofrequency fields of wireless smart meters in Victoria, Australia: A case series. Altern. Ther. Health Med..

[9] Thomee S., Harenstam A., Hagberg M. (2011). Mobile phone use and stress, sleep disturbances, and symptoms of depression among young adults: A prospective cohort study. BMC Public Health.

[10] Wang K., Lu J., Xing Z., Zhao Q., Hu L., Xue L., Zhang J., Mei Y. (2017). Effect of 1.8 GHz radiofrequency electromagnetic radiation on novel object associative recognition memory in mice. Sci. Rep..

[11] Bouji M., Lecomte A., Gamez C., Blazy K., Villégier A. (2016). Neurobiological effects of repeated radiofrequency exposures in male senescent rats. Biogerontology.

[12] Arendash G.W., Sanchez-Ramos J., Mori T., Mamcarz M., Lin X., Runfeldt M., Wang L., Zhang G., Sava V., Tan J. (2010). Electromagnetic field treatment protects against and reverses cognitive impairment in Alzheimer’s disease mice. J. Alzheimers Dis..

[13] Aldad T.S., Gan G., Gao X., Taylor H.S. (2012). Fetal radiofrequency radiation exposure from 800–1900 MHz-Rated cellular telephones affects neurodevelopment and behavior in mice. Sci. Rep..

[14] Daniels W.M.U., Pitout I.L., Afullo T.J.O., Mabandla M.V. (2009). The effect of electromagnetic radiation in the mobile phone range on the behaviour of the rat. Metab. Brain Dis..

[15] Can A., Dao D.T., Terrillion C.E., Piantadosi S.C., Bhat S., Gould T.D. (2012). The tail suspension test. J. Vis. Exp..

[16] Can A., Dao D.T., Arad M., Terrillion C.E., Piantadosi S.C., Gould T.D. (2012). The mouse forced swim test. J. Vis. Exp..

[17] Vorhees C.V., Williams M.T. (2006). Morris water maze: Procedures for assessing spatial and related forms of learning and memory. Nat. Protoc..

[18] Ju G., Zhao X. (2012). Practical Experimental Techniques in Neurobiology.

[19] Sari Y., Hammad L.A., Saleh M.M., Rebec G.V., Mechref Y. (2010). Alteration of selective neurotransmitters in fetal brains of prenatally alcohol-treated C57BL/6 mice: Quantitative analysis using liquid chromatography/tandem mass spectrometry. Int. J. Dev. Neurosci..

[20] Kumar M., Singh S.P., Chaturvedi C.M. (2016). Chronic nonmodulated microwave radiations in mice produce anxiety-like and depression-like behaviours and calcium- and no-related biochemical changes in the brain. Exp. Neurobiol..

[21] Zhang Y., Li Z., Gao Y., Zhang C. (2015). Effects of fetal microwave radiation exposure on offspring behavior in mice. J. Radiat. Res..

[22] Kumlin T., Iivonen H., Miettinen P., Juvonen A., van Groen T., Puranen L., Pitkaaho R., Juutilainen J., Tanila H. (2007). Mobile phone radiation and the developing brain: Behavioral and morphological effects in juvenile rats. Radiat Res..

[23] Júnior L.C.D.C., Guimarães E.D.S.G., Musso C.M., Stabler C.T., Garcia R.M.G., Mourão-Júnior C.A., Andreazzi A.E. (2014). Behavior and memory evaluation of Wistar rats exposed to 1.8 GHz radiofrequency electromagnetic radiation. Neurol. Res..

[24] Barthélémy A., Mouchard A., Bouji M., Blazy K., Puigsegur R., Villégier A. (2016). Glial markers and emotional memory in rats following acute cerebral radiofrequency exposures. Environ. Sci. Pollut. Res..

[25] Saikhedkar N., Bhatnagar M., Jain A., Sukhwal P., Sharma C., Jaiswal N. (2014). Effects of mobile phone radiation (900 MHz radiofrequency) on structure and functions of rat brain. Neurol. Res..

[26] Morris R. (1984). Developments of a water-maze procedure for studying spatial learning in the rat. J. Neurosci. Methods.

[27] Sienkiewicz Z.J., Blackwell R.P., Haylock R.G., Saunders R.D., Cobb B.L. (2000). Low-level exposure to pulsed 900 MHz microwave radiation does not cause deficits in the performance of a spatial learning task in mice. Bioelectromagnetics.

[28] Klose M., Grote K., Spathmann O., Streckert J., Clemens M., Hansen V.W., Lerchl A. (2014). Effects of early-onset radiofrequency electromagnetic field exposure (GSM 900 MHz) on behavior and memory in rats. J. Vis. Exp..

[29] Jeong Y.J., Kang G.Y., Kwon J.H., Choi H.D., Pack J.K., Kim N., Lee Y.S., Lee H.J. (2015). 1950 MHz electromagnetic fields ameliorate Aβ pathology in Alzheimer’s disease mice. Curr. Alzheimer. Res..

[30] Belzung C., Turiault M., Griebel G. (2014). Optogenetics to study the circuits of fear- and depression-like behaviors: A critical analysis. Pharmacol. Biochem. Behav..

[31] Meyer-Lindenberg A. (2010). Behavioural neuroscience: Genes and the anxious brain. Nature.

[32] Bird C.M., Burgess N. (2008). The hippocampus and memory: Insights from spatial processing. Nat. Rev. Neurosci..

[33] Heresco-Levy U. (2003). Glutamatergic neurotransmission modulation and the mechanisms of antipsychotic atypicality. Prog. Neuro-Psychopharmacol. Biol. Psychiatry.

[34] HYND M. (2004). Glutamate-mediated excitotoxicity and neurodegeneration in Alzheimer’s disease. Neurochem. Int..

[35] Millan M.J. (2003). The neurobiology and control of anxious states. Prog. Neurobiol..

[36] Cavalcante G.I., Chaves F.A., Linhares M.I., de Carvalho L.C., Venancio E.T., Rios E.R., de Souza F.C., Vasconcelos S.M., Macedo D., de Franca F.M. (2017). HIV antiretroviral drug Efavirenz induces anxiety-like and depression-like behavior in rats: evaluation of neurotransmitter alterations in the striatum. Eur. J. Pharmacol..

[37] Li Y., Wang D., Peng R., Li Z., Dong B., Dong F., Liang Y., Hu W. (2003). Effects of electromagnetic pulse on contents of amino acids in hippocampus of rats. Chin. J. Ind. Hyg. Occup. Dis..

[38] Blokland A. (1995). Acetylcholine: A neurotransmitter for learning and memory. Brain Res. Rev..

[39] Winkler J., Suhr S.T., Gage F.H., Thal L.J., Fisher L.J. (1995). Essential role of neocortical acetylcholine in spatial memory. Nature.

[40] Lydiard R.B. (2003). The role of GABA in anxiety disorders. J. Clin. Psychiatry.

[41] Nemeroff C.B. (2003). The role of GABA in the pathophysiology and treatment of anxiety disorders. Psychopharmacol. Bull..

[42] Olexová L., Štefánik P., Kršková L. (2016). Increased anxiety-like behaviour and altered GABAergic system in the amygdala and cerebellum of VPA rats—An animal model of autism. Neurosci. Lett..

[43] Zhang W., Zhao R., Li X., Cui X., Zhao Z., Mao Y., Wu F., Tang Q. (2016). Effect of Yi-nao-jie-yu decoction on γ-aminobutyric acid type A receptor in the hippocampus and serum inflammatory factors in a rat model of post stroke anxiety. Neuropsychiatric Dis. Treat..

[44] International Commission on Non-Ionizing Radiation Protection (1998). Guidelines for limiting exposure to time-varying electric magnetic and electromagnetic fields (up to 300 GHz). Health Phys..

[45] Institute of Electrical and Electronics Engineers (2006). IEEE Standard for Safety Levels with Respect to Human Exposure to Radio Frequency Electromagnetic Fields, 3 kHz to 300 GHz, IEEE Std C95.1™-2005.

